# Behavioral Changes Following Tirzepatide Treatment in Prader–Willi Syndrome: A Case Report

**DOI:** 10.1192/j.eurpsy.2026.11096

**Published:** 2026-07-31

**Authors:** A. Pavesic Radonja, J. Batur, A. Čubranić Dominković, S. Blazevic Zelic

**Affiliations:** 1 Department of psychiatry, Clinical hospital Center Rijeka; 2 Faculty of Medicine, University of Rijeka, Rijeka, Croatia

## Abstract

**Introduction:**

Prader–Willi syndrome (PWS) is a rare genetic disorder caused by the absence of paternal gene expression on chromosome 15q11–q13. It is characterized by early hypotonia, hyperphagia leading to obesity, endocrine dysfunction, and varying degrees of cognitive impairment. Individuals with PWS frequently exhibit behavioral and psychological problems, such as temper outbursts, compulsivity, and mood instability, which often contribute to hospitalizations. Obesity-related complications, including hypoventilation and recurrent respiratory infections, further increase morbidity and reduce quality of life. Obesity is usually severe, and one of the newly available treatment options is tirzepatide, a dual glucose-dependent insulinotropic polypeptide (GIP) and glucagon-like peptide-1 (GLP-1) receptor agonist that improves glycemic control and promotes weight loss in patients with type 2 diabetes and obesity.

**Objectives:**

This report aims to present the case of a 21-year-old male patient with Prader–Willi syndrome, highlighting the complexity of pharmacotherapy due to his sensitivity to side effects, and to raise concern about the potential aggravation of aggressive symptoms following the introduction of a GIP/GLP-1 agonist.

**Methods:**

Case report

**Results:**

The patient was admitted to our Psychiatric Clinic several times over the past two years due to behavioral issues. His medical history included obesity, hypoventilation syndrome, and recurrent lower respiratory tract infections. According to the heteroanamnestic report, the patient’s psychological condition has deteriorated since the initiation of tirzepatide in April 2025. He displayed recurrent temper outbursts, increased irritability, and more frequent episodes of aggression. The aggressive episodes with agitation provoked hypoventilation, which, in combination with psychopharmacological treatment during hospitalization, contributed to the development of respiratory complications. A pharmacological evaluation was performed to assess the possible association between the introduction of tirzepatide and the worsening of behavior. Following the recommendation of the clinical pharmacologist, pharmacogenetic testing was indicated.

**Conclusions:**

This case highlights the need for careful coordination of care between endocrinologists, psychiatrists, and clinical pharmacologists in patients with Prader–Willi syndrome. Early recognition of behavioral changes and potential drug-related effects is essential to prevent complications and reduce the risk of hospitalization.

**Disclosure of Interest:**

None Declared

